# Dual-clamp technique facilitating end-to-side suture anastomosis in murine cervical heart transplantation

**DOI:** 10.3389/ti.2026.17404

**Published:** 2026-09-01

**Authors:** Xia Huang, Xuechun Zhao, Zipei Wang

**Affiliations:** 1 Institute of Organ Transplantation, Tongji Hospital, Tongji Medical College, Huazhong University of Science and Technology, Key Laboratory of Organ Transplantation, Ministry of Education, NHC Key Laboratory of Organ Transplantation, Key Laboratory of Organ Transplantation, Chinese Academy of Medical Sciences, Organ Transplantation Clinical Medical Research Center of Hubei Province, Wuhan, China; 2 Division of Hepato-Pancreato-Biliary Surgery, Tongji Hospital, Tongji Medical College, Huazhong University of Science and Technology, Wuhan, China

**Keywords:** end-to-side anastomosis, heart transplantation, microsurgical technique, mouse model, transplant model

Dear Editors,

Heterotopic cervical heart transplantation (HT) in mice is a widely used preclinical model in transplant immunology and chronic allograft vasculopathy research [1, 2]. Although the cuff technique is popular due to its simplicity, it compromises unilateral cerebral blood flow, requires custom-made cuffs, and carries a higher risk of thrombosis and vessel twisting. At our center, we favor manual end-to-side suture anastomosis, as it preserves the continuity of the common carotid artery (CCA) and external jugular vein (EJV), thereby preventing neurological complications and adhering to the 3Rs principle. Moreover, this technique avoids high-velocity arterial jets in the end-to-end grafts and ensures maximum comparability between studies using the abdominal HT model [3].

A major technical hurdle of this manual approach is that the CCA lies deep within the body and is widely separated from the EJV, making precise suturing highly challenging, especially in the confined cervical space. To overcome this, we apply a dual-clamp occlusion technique [4] (Figure 1A). Two microclamps are used: one placed at the proximal end and one at the distal end, each simultaneously clamping both the CCA and the EJV, physically bringing the two vessels into close proximity and effectively eliminating height mismatch (Figures 1B–E). Additionally, sterile, nonwoven pads moistened with cold saline are placed over the clamp handles. The gentle downward pressure not only keeps the graft cool but also elevates the deeper CCA to the superficial plane. This simple modification makes the artery-first anastomosis and the entire everting suture feasible.

**FIGURE 1:**
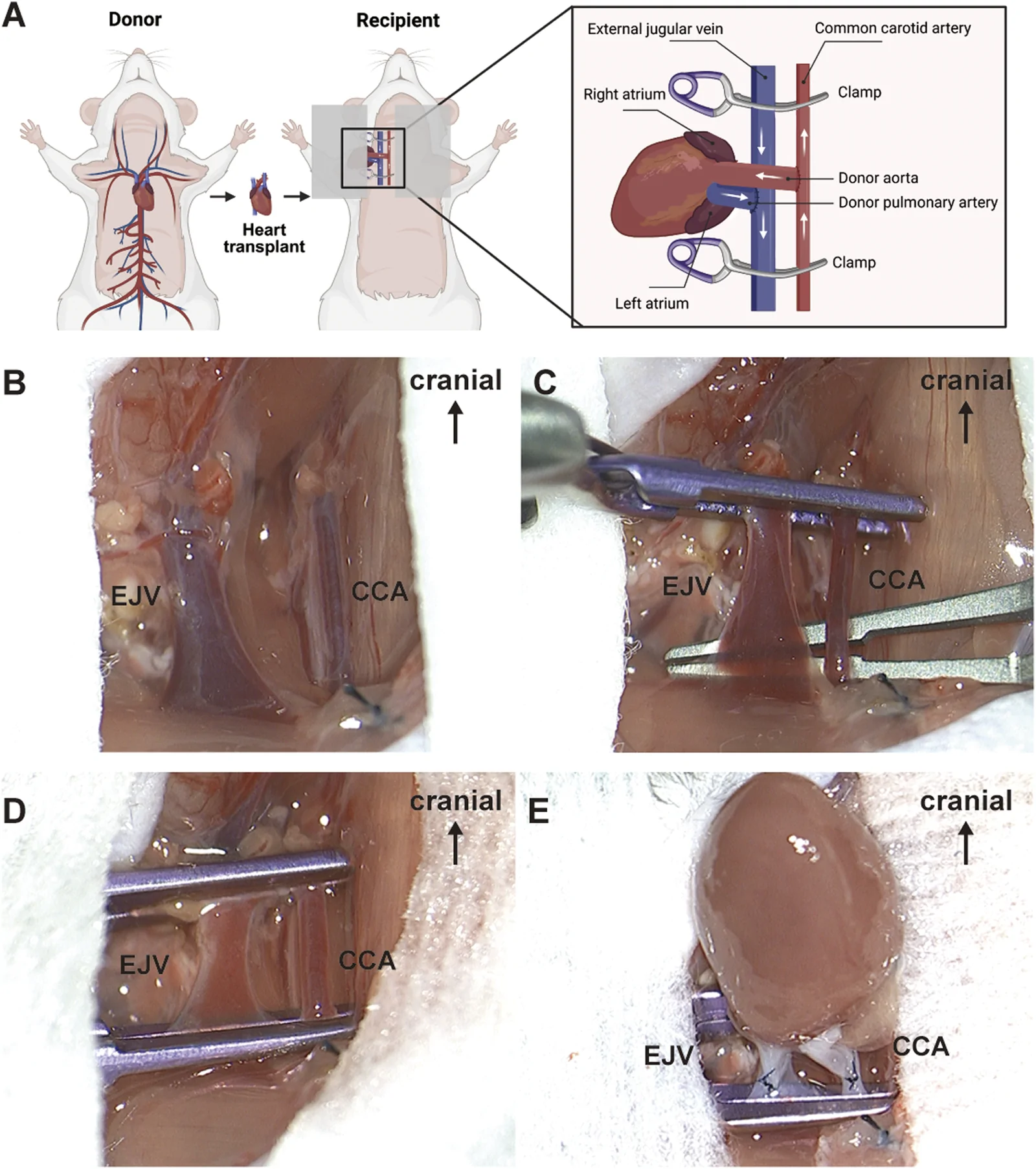
**(A)** Schematic diagram of end-to-side anastomosis using the dual-clamp vascular occlusion technique. **(B)** Isolate the recipient’s CCA and EJV. **(C)** Clamp the CCA and EJV simultaneously with a microvascular clamp on the cranial side. **(D)** Clamp the CCA and EJV simultaneously with a microvascular clamp on the caudal side. **(E)** View of the allograft heart after completing all anastomoses. CCA, common carotid artery. EJV, external jugular vein.

Since implementing this refined technique in our established HT program (>1,000 cases since 2010), we have achieved a technical success rate of 83/87 (95.4%) in recent consecutive procedures. Technical success was defined as graft survival with maintained palpable contractility throughout the 100-day observation period (assessed daily by palpation). All 83 successful grafts remained functional during this period, free of thrombosis and other vascular complications. Although manual suturing is generally considered more technically demanding than the cuff method, the total operative time for the recipient (∼25–30 min) was not prolonged. In fact, the anastomosis itself (15–20 min) was faster than cuff installation (20–30 min) [5]. In our institutional training curriculum for microsurgical transplantation, three surgeons with prior abdominal HT experience achieved consistent success in approximately 3 to 5 procedures, whereas three trainees without a microsurgical background required 50 to 70 operations to become proficient.

While we acknowledge the single-center nature of this study and the lack of a prospective comparison with the cuff technique, we believe this technical modification provides a valuable refinement for cervical HT, particularly for microsurgeons with prior abdominal experience transitioning to the cervical model.

## Data Availability

The original contributions presented in the study are included in the article/supplementary material, further inquiries can be directed to the corresponding author.
